# Type 1-skewed neuroinflammation and vascular damage associated with *Orientia tsutsugamushi* infection in mice

**DOI:** 10.1371/journal.pntd.0005765

**Published:** 2017-07-24

**Authors:** Lynn Soong, Thomas R. Shelite, Yan Xing, Harica Kodakandla, Yuejin Liang, Brandon J. Trent, Paulina Horton, Kathryn C. Smith, Zhenyang Zhao, Jiaren Sun, Donald H. Bouyer, Jiyang Cai

**Affiliations:** 1 Department of Microbiology and Immunology, Institute of Human Infections and Immunity, University of Texas Medical Branch, Galveston, Texas, United States of America; 2 Department of Pathology, University of Texas Medical Branch, Galveston, Texas, United States of America; 3 Department of Internal Medicine/Division of Infectious Diseases, University of Texas Medical Branch, Galveston, Texas, United States of America; 4 Pediatrics Department, People's Hospital of Henan Province, Zheng Zhou, Henan, China; 5 School of Medicine, University of Texas Medical Branch, Galveston, Texas, United States of America; 6 Center in Environmental Toxicology, University of Texas Medical Branch, Galveston, Texas, United States of America; 7 Department of Ophthalmology, Institute of Human Infections and Immunity, University of Texas Medical Branch, Galveston, Texas, United States of America; University of California San Diego School of Medicine, UNITED STATES

## Abstract

**Background:**

Scrub typhus is a life-threatening disease, due to infection with *O*. *tsutsugamushi*, a Gram-negative bacterium that preferentially replicates in endothelial cells and professional phagocytes. Meningoencephalitis has been reported in scrub typhus patients and experimentally-infected animals; however, the neurological manifestation and its underlying mechanisms remain poorly understood. To address this issue, we focused on *Orientia tsutsugamushi* Karp strain (OtK), and examined host responses in the brain during lethal versus self-healing scrub typhus disease in our newly established murine models.

**Principle findings:**

Following inoculation with a lethal dose of OtK, mice had a significant increase in brain transcripts related to pathogen-pattern recognition receptors (TLR2, TLR4, TLR9), type-1 responses (IFN-γ, TNF-α, CXCL9, CXCR3), and endothelial stress/damage such as angiopoietins, but a rapid down-regulation of Tie2. Sublethal infection displayed similar trends, implying the development of type 1-skewed proinflammatory responses in infected brains, independent of time and disease outcomes. Focal hemorrhagic lesions and meningitis were evident in both infection groups, but pathological changes were more diffuse and frequent in lethal infection. At 6–10 days of lethal infection, the cortex and cerebellum sections had increased ICAM-1-positive staining in vascular cells, as well as increased detection of CD45^+^ leukocytes, CD3^+^ T cells, IBA1^+^ phagocytes, and GFAP^+^ astrocytes, but a marked loss of occludin-positive tight junction staining, implying progressive endothelial activation/damage and cellular recruitment in inflamed brains. *Orientia* were sparse in the brains, but readily detectable within lectin^+^ vascular and IBA-1^+^ phagocytic cells. These CNS alterations were consistent with type 1-skewed, IL-13-suppressed responses in lethally-infected mouse lungs.

**Significance:**

This is the first report of type 1-skewed neuroinflammation and cellular activation, accompanied with vascular activation/damage, during OtK infection in C57BL/6 mice. This study not only enhances our understanding of the pathophysiological mechanisms of scrub typhus, but also correlates the impact of immune and vascular dysfunction on disease pathogenesis.

## Author summary

Scrub typhus is a life-threatening disease, due to infection with *O*. *tsutsugamushi*, a Gram-negative bacterium that preferentially replicates in endothelial cells and professional phagocytes. Meningoencephalitis has been reported in scrub typhus patients and in experimentally infected animals; however, the neurological manifestation and its underlying mechanisms remain poorly understood. In this study, we utilized our established mouse models of severe and self-healing scrub typhus and revealed infection dose-dependent alterations in the CNS. Following lethal and sublethal doses of infection with *O*. *tsutsugamushi*, infected brains showed strong expression of genes for proinflammatory cytokines and mediators, as well as increased activation of endothelial cells and microglial cells/monocytes, accompanied with foci of vascular damage. Importantly, vascular leakage and inflammation in the CNS of infected mice were positively linked to the selective activation of type 1 cytokines, but suppression of type 2 cytokines, in inflamed lung tissues. Together, our data reveal type 1-skewed neuroinflammation and foci vascular leakage in the brain, mostly due to immune dysregulation and acute damage in the peripheral organs. This study improves our understanding of disease mechanisms and opens new avenues for the exploration of treatment options.

## Introduction

*Orientia tsutsugamushi* is the etiological agent for scrub typhus, a human disease highly endemic in the “tsutsugamushi triangle” that expands a broad geographic region in Southeast Asia. Approximately one million new cases appear annually, and one billion people are at risk of infection. The incidence of scrub typhus cases has been increasing in recent years, and new cases are reported in other geographic areas, including Africa and South America [1]. While antibiotics such as doxycycline and ciprofloxacin are known to be effective for treatment, missed or delayed diagnosis and persistent infection are major issues, among others, for this neglected tropical disease [2].

After transmission to humans by chiggers, some patients may exhibit eschar at the inoculation site, followed by fever, skin rash, and non-specific flu-like symptoms [3]. The hematogenous spread of bacteria via endothelial cells (EC) and/or macrophages to visceral organs can give rise to organ-specific inflammation patterns, tissue damage, and multi-organ failure. Scrub typhus has diverse clinical manifestations, ranging from a non-specific febrile illness to severe multi-organ dysfunction, with mortality up to 30% in untreated patients [3]. Disease severity and mortality are associated with increased endothelial and macrophage markers [4]. CNS involvement is common among severe cases, and diverse neurological symptoms, including headache, vomiting, altered sensorium, seizures, have been reported [5,6,7,8,9]. Patients can be categorized into meningitis, encephalopathy, and encephalitis, based on neck stiffness, consciousness level, and cerebrospinal fluid cell count, as well as other pathological evidence of focal neurological deficits [10,11,12]. Dittrich *et al*. recently reported that *Orientia* and *Rickettsia sp*. account for 9% of all CNS infections in Laos [13]. A cross-sectional study in north India has recently shown that two-thirds of severely ill scrub typhus patients (23/37) have menigoencephalitis or encephalopathy, with CNS involvement mostly presented after 10 days of disease, suggesting the inclusion of scrub typhus in the differential diagnosis of febrile encephalopathy [14]. However, there is a paucity of studies examining the molecular basis of the neurological manifestations of scrub typhus.

The severity of scrub typhus is considered to be dependent on host immune status and the *Orientia* strains involved. OtK is the most prevalent strain in human patients, accounting for approximately 50% infections in endemic countries [1]. OtK is also the most virulent *Orientia* strain in experimental animals and can cause lethal or sublethal infections in outbred and inbred mice, depending on the inoculation doses and routes. A recent report by Keller and colleagues [15] has provided solid evidence for dissemination of OtK from footpad inoculation site in BALB/c mice to draining lymph nodes and to visceral organs such as the lungs and brains. Using this self-limiting model of scrub typhus, these authors have documented the kinetics of bacterial dissemination in the context of macrophage/astrocyte activation in the CNS, implying a breakdown of the blood-brain barrier during disease progression. Our group also developed an i.d. inoculation model in C57BL/6 mouse ears for kinetic studies of bacterial dissemination and cellular and antibody responses at acute versus persistent stages of OtK infection [16]. While these models mimic natural infection routes, they have intrinsic limitations. For example, while these skin-inoculation models are useful for studying self-limiting or non-severe scrub typhus, they do not resemble pathogenesis of severe disease or vascular dysfunction during lethal infection.

Our newly developed, severe scrub typhus models for endothelium-targeted OtK infection in C57BL/6 mice have offered additional values for the study of host-OtK interaction, as it permitted mechanistic examination of pathogenesis during lethal versus sublethal infections [17,18]. Comparative studies in wild-type mice, knockout mice lacking a given host gene such as alarmin IL-33, and *in vitro* infection in human EC cultures have revealed tissue- and EC-specific alterations [19]. These studies have discovered that type 1-skewed inflammatory responses are common features in OtK-infected lungs, liver, and spleens, and that releasing endogenous danger signals contributes to scrub typhus pathogenesis. However, it remains less clear as to the magnitude of CNS alterations, or their association with systematic immune alterations, during lethal versus sublethal infections.

We hypothesize that neuroinflammation and vascular leakage in the brain is closely associated with type 1-skewed inflammation during the early stages of *O*. *tsutsugamushi* infection. Using our endothelium-targeted OtK infection models, we have revealed, herein, the kinetics of gene up-regulation for immune recognition and inflammatory responses in the brain tissues. Our novel findings in the brains of lethally-infected mice include 1) a significant increase in type-1 immune markers and vascular destabilizing factors angiopoietins 2 and 1 (Ang2 and Ang2/1 ratio), 2) a marked activation of EC and macrophages/astrocytes with sparse colocation of OtK in these cells, and 3) severe loss of Tie2 (an endothelial tyrosine kinase receptor) and tight junctions. Our findings support a notion of vascular activation and dysfunction during this infection and provide new clues for further examination of tissue-specific immune mechanisms.

## Materials and methods

### Ethics statement

Tissue processing and analysis procedures were performed in the BSL2 or BSL3 facilities, as approved by the Institutional Biosafety Committee, in accordance with Guidelines for Biosafety in Microbiological and Biomedical Laboratories. UTMB operates to comply with the USDA Animal Welfare Act (Public Law 89–544), the Health Research Extension Act of 1985 (Public Law 99–158), the Public Health Service Policy on Humane Care and Use of Laboratory Animals, and the NAS Guide for the Care and Use of Laboratory Animals (ISBN-13). UTMB is a registered Research Facility under the Animal Welfare Act and has a current assurance on file with the Office of Laboratory Animal Welfare, in compliance with NIH Policy. *Orientia tsutsugamushi* Karp strain (unknown passage history) was obtained from the Rickettsial and Ehrlichial Species Collection at the University of Texas Medical Branch courtesy of Nicole L. Mendell and David H. Walker.

### Mouse infection

Female C57BL/6 mice (Envigo RMS, Inc. or Jackson Lab) were maintained under specific pathogen-free conditions and used at 8- to 12-weeks of age following protocols approved by the Institutional Animal Care and Use Committee (protocol # 1302003) at the University of Texas Medical Branch (UTMB). All infection studies were performed in an on-campus ABSL3 facility in the Galveston National Laboratory; mice (5 per group) were infected with the same OtK stocks that were prepared from mouse liver extracts, as in our reports [17,18]. For lethal infection, mice were inoculated i.v. with 1.0 x 10^6^ focus-forming units (FFU) of OtK in 200 μl or with PBS (mock) and monitored daily; tissues were collected at 2, 6, and 10 days. For sublethal infection, mice were inoculated i.v. with 1.0X10^4^ FFU, monitored daily, and euthanized between days 12–14 post-infection.

### Quantitative RT-PCR analysis

Tissues were collected in an RNA*Later* solution. Brain total RNA was extracted via TRIzol in combination with the Qiagen RNeasy kit. Specific primers for qRT-PCR are listed in our previous reports [18,19]; others (TLR2, TLR4, TLR9, CXCR3, and Tie2) are provided in **S1 Table**. Relative abundance of transcripts was calculated by using the 2^-ΔΔCT^ method and compared to housekeeping genes glyceraldehyde-3-phosphate dehydrogenase (GAPDH) or β-actin.

#### Histological evaluations

Brain and lung tissues were fixed in 10% formalin and embedded in paraffin; 5-μm sections were stained with hematoxylin and eosin (H&E). For quantification, 10 images from both the cortex and cerebellum regions were taken under an Olympus BX53 microscope. Lesions per 10x field-of-view (hemorrhages, vascular occlusions, typhus nodules, and mengioencephalitic infiltrates) were counted by different observers. The counts of individual mice were pooled, averaged, and presented as “number of lesions per animal.”

### Immunofluorescence microscopy assay

Brain tissues were fixed in 4% paraformaldehyde (EMS, Hatfield, PA, USA) in 5% sucrose/PBS for 2 days at 4°C and switched to a fresh fixing solution at 4°C overnight. Tissues were transferred into 20% sucrose/PBS for 8 h at 4°C and frozen in O.C.T. compound (TissueKek, Sakura Finetek, Torrance, USA). Sections (8-μm) were processed in a humidified black box for fixing (with pre-chilled acetone for 10 min) and washing (with ddH_2_O for 5 times; TBS-0.025% Triton twice). After blocking, sections were incubated at 4°C overnight with 1:50–1:200 diluted rat or rabbit Abs: anti-ICAM-1 (Abcam, Cambridge, MA, USA), anti-CD3 (BioLegend, San Diego, CA, USA), anti-CD45 (BD Bioscience, San Jose, CA, USA), anti-IBA-1 (ionised calcium binding adapter molecule-1, a microglia/macrophage marker, Wako, Osaka, Japan), anti-GFAP (glial fibrillary acidic protein, an astrocyte-specific marker, Cell Signaling Technology, Boston, MA, USA), anti-occludin (a tight junction marker, Abcam). Next, Alexa Fluor 488- or 555-conjugated, goat anti-rat or anti-rabbit IgG (H+L, 1:1,000–1:2,000 dilutions, Life Technologies, Grand Island, NY, USA) were applied for 2 h at room temperature. For staining infected EC or phagocytes, 1:100 diluted FITC-conjugated *Griffonia Simplicifolia* lectin I (isolectin B4, Vector Lab, Burlingame, CA, USA) [20] or rat anti-IBA-1 was used together with 1:500-diluted rabbit anti-OtK Ab [17,18]. Lastly, sections were incubated in DAPI (1:5,000, Sigma-Aldrich, St. Louis, MO, USA) for 5 min and washed thoroughly. Infected sections stained with secondary Abs and DAPI only served as negative controls to optimize staining conditions. For each section, at least 6 low- and 6 high-magnification fields of the cortex and cerebellum were imaged on a Carl Zeiss Axio Observer fluorescence microscope equipped with ApoTome and Zen imaging software. Acquisition settings were identical among samples of different experimental groups. Representative images at each time point are presented.

### Bio-plex assay

For cytokine profiles, lung tissue homogenates (50 μg proteins each) were measured in duplicates by using Procarta Plex Mouse Cytokine Panel (eBioscience, San Diego, CA, USA), as in our previous report [19]. Raw data were acquired as the relative fluorescence intensity and then converted to the concentration, according to the standard curves.

### Statistical analysis

Prism 6 (GraphPad Software) was used for data representations and statistical calculations. The unpaired Student’s t test and the Mann-Whitney test were used for determining statistical significance. Statistically significant values are referred to as *, *p* < 0.05; **, *p* < 0.01, ***, *p* < 0.001, respectively.

## Results

### Type 1-skewed immune responses and endothelial dysfunction in mouse brains following lethal or sublethal infection with *O*. *tsutsugamushi*

**Fig 1:**
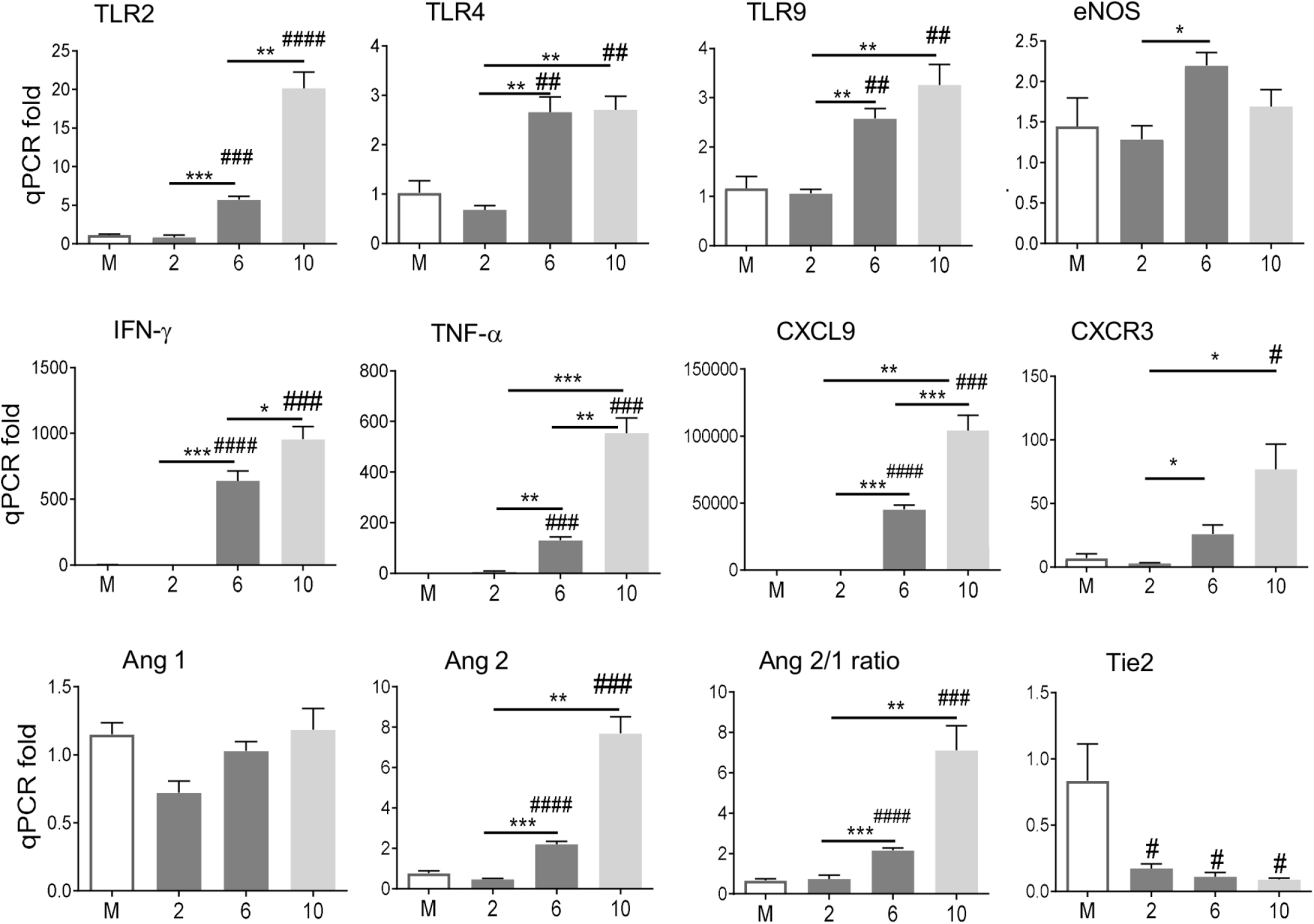
Brain gene expression profiles following lethal infection with *O*. *tsutsugamushi*. C57BL/6 mice (5/group) were inoculated i.v. with *O*. *tsutsugamushi* Karp strain (1.0X10^6^ FFU) or with PBS (mock). Total RNAs were extracted from brain tissues at indicated days of infection for qRT-PCR analyses. Data are shown as mean in each group and are presented as “qPCR fold” after normalization to housekeeping gene (GAPDH) and statistically compared using the Student’s t-test; #, *p* < 0.05; ##, *p* < 0.01, ###, *p* < 0.001 (compared with the mock groups); *, *p* < 0.05; **, *p* < 0.01, ***, *p* < 0.001 (compared with marked groups). Representative data from two independent experiments with similar trends are shown.

C57BL/6 mice are highly susceptible to OtK infection and develop acute/lethal disease or persistent infection, depending on inoculation doses and routes [16,17,19]. Here, we focused on the i.v. route inoculation and infection-associated alterations in the brain. Mice receiving a lethal-dose of OtK started losing weight at day 6, reached weight-loss peak on day 10, and expired (100%) between days 12–13 (**S1A Fig**), presumably due to extensive proinflammatory responses and acute tissue damage [18,19]. To define host responses in the CNS, we examined expression levels of transcripts that represent innate, type-1, or type-2 responses, and endothelial function, respectively. We found no major changes at day 2 (the incubation period), but day 6 samples showed significant increase for a panel of genes related to pattern-recognition receptors (TLR2, TLR4, TLR9), type-1 responses (IFN-γ, TNF-α, CXCL9, CXCR3), and endothelial stress or damage such as endothelial nitric oxide synthase (eNOS), Ang2 and Ang2/1 ratio, respectively (**Fig 1**). Nearly all of these biomarkers reached their expression peaks at day 10. In sharp contrast, Tie2 expression, an endothelial tyrosine kinase receptor that regulates angiogenesis and supports the integrity of endothelial junctions [21,22], was rapidly down-regulated at day 2 and reached its lowest levels at day 10. There were no detectable levels of IL-4, a signature type-2 cytokine, in any of our tested samples. These data suggest a type 1-skewed, proinflammatory gene profile, accompanied with endothelial dysfunction, in the brains of mice following infection with a lethal-dose of *O*. *tsutsugamushi*.

**Fig 2:**
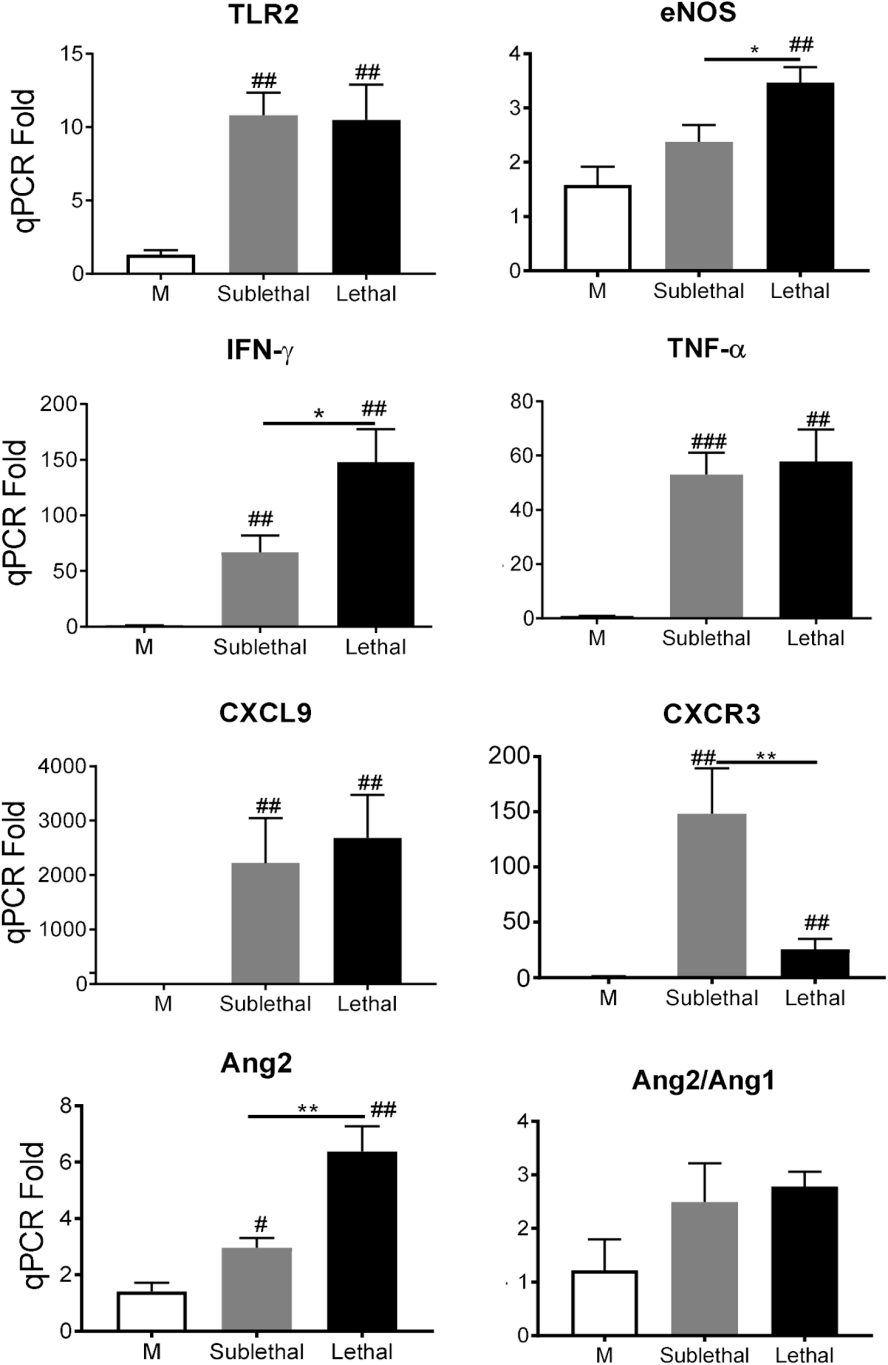
Gene expression profiles in the brain following sub-lethal versus lethal infections. Mice (5/group) were inoculated i.v. with either *O*. *tsutsugamushi* Karp strain at a sublethal dose (1.0X10^4^ FFU), a lethal dose (1.0X10^6^ FFU), or with PBS (mock). Brain total RNAs were extracted from sublethal infection (day 12) and lethal infection (day 10) for qRT-PCR analyses. Data are shown as mean in each group and are presented as “qPCR fold” after normalization to housekeeping gene (GAPDH) and statistically compared using the Student’s t-test; #, *p* < 0.05; ##, *p* < 0.01, ###, *p* < 0.001 (compared with mock groups); *, *p* < 0.05; **, *p* < 0.01 (compared with marked groups). Representative data from two independent experiments with similar trends are shown.

To validate and expand these findings, we next examined mice with sublethal infection, in which all mice survived, but their body weight remained lower than mock controls at the time of tissue collection between 12–14 days (**S1B Fig**). Compared with PBS controls, sublethal infection also markedly induced mRNA levels of TLR2, IFN-γ, TNF-α, CXCL9, CXCR3, and Ang2 (**Fig 2**). Among tested markers, eNOS, IFN-γ, and Ang2 were significantly higher in lethal than in sublethal groups. Together, our data indicate that the i.v. route of inoculation resulted in type 1-skewed proinflammatory responses in the brains of OtK-infected mice, regardless of the infection doses and disease outcomes.

### Brain histopathology and cellular responses following lethal and sublethal infections

**Fig 3:**
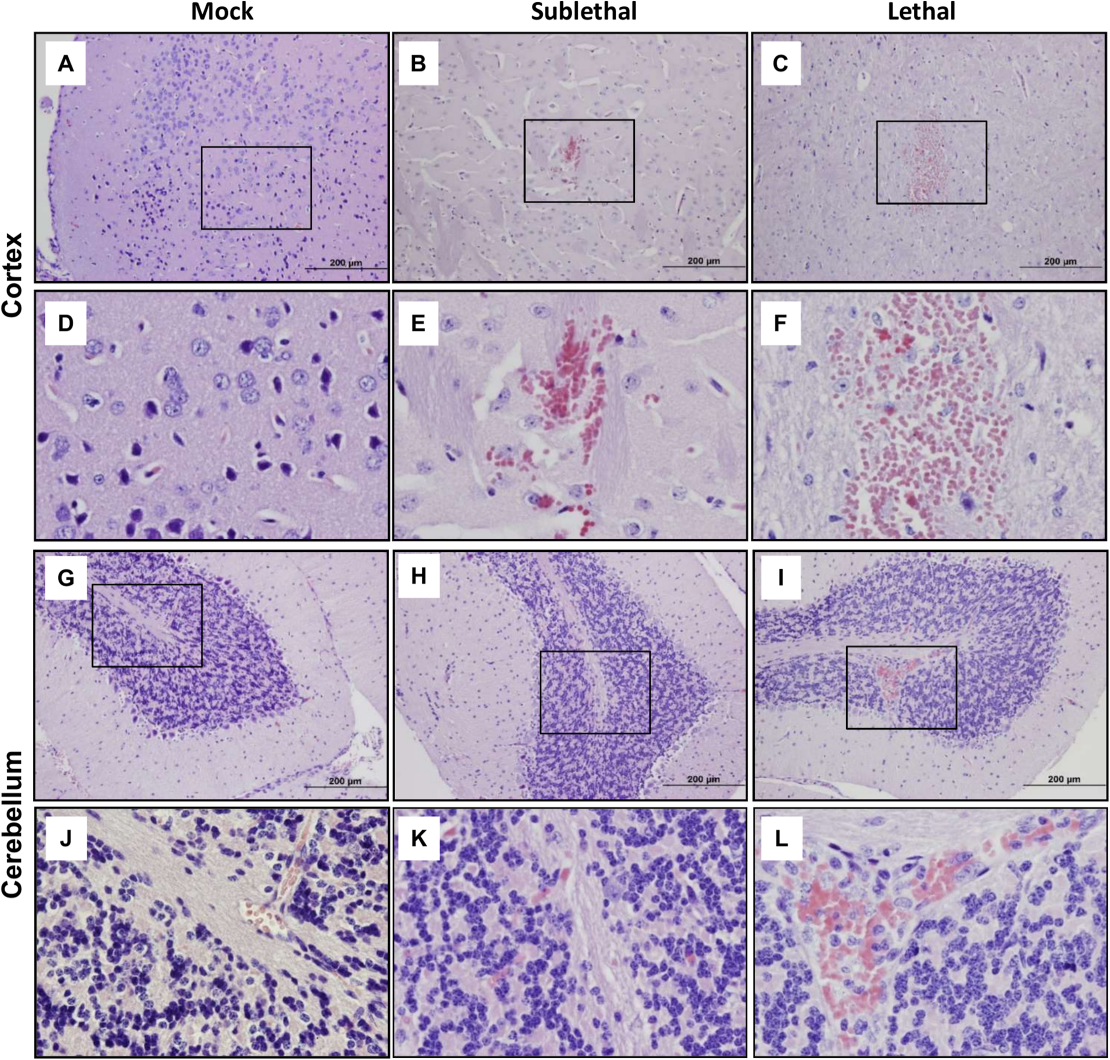
Focal hemorrhages in *Orientia*-infected brain tissues. Mice (5/group) were inoculated with *O*. *tsutsugamushi* or PBS, as described in *Fig 2*. Brain tissues were collected from sublethal infection (day 12) and lethal infection (day 10) and fixed in 10% formalin. Tissue sections were stained with H&E. Images of brain cortex and cerebellum were photographed at 10X (A-C and G-I), and their corresponding boxed areas were photographed at 40X (D-F and J-L), respectively.

H&E staining indicated focal lesions (hemorrhages, vascular occlusions, typhus nodules, and mengioencephalitic infiltrates) in the cortex sections of both lethal and sublethal infection groups (**Fig 3**). Lethally-inoculated mice tended to have more diffuse and frequent hemorrhages and meningitis (at day 10, Fig 3C and 3F) compared with sublethal infection (days 12–14). Similar trends were observed for the cerebellum sections (Fig 3I and 3L). Brains of mock controls showed no hemorrhages or occlusion. Lethal inoculation resulted in a 4-fold increase in cortex and cerebellum lesions per animal than did sublethal inoculation (**S2 Fig**). The magnitude of meningitis and cerebral perivascular infiltrates in these infected groups positively correlated with the levels of pathological changes in the lungs (**S3 Fig**). The lungs of sublethal infection displayed foci of lesions, characterized by cellular infiltrates, thickening of interstitium, and minor consolidation of the alveoli. The pathological changes in lethally-infected lungs, however, were more severe and diffuse. While some regions displayed cellular infiltrates, interstitium thickening, and tissue damage (circle, S2E Fig), some alveoli diffusely filled with exudate, indicating severe vascular leakage in lethal infection (S2F Fig).

**Fig 4:**
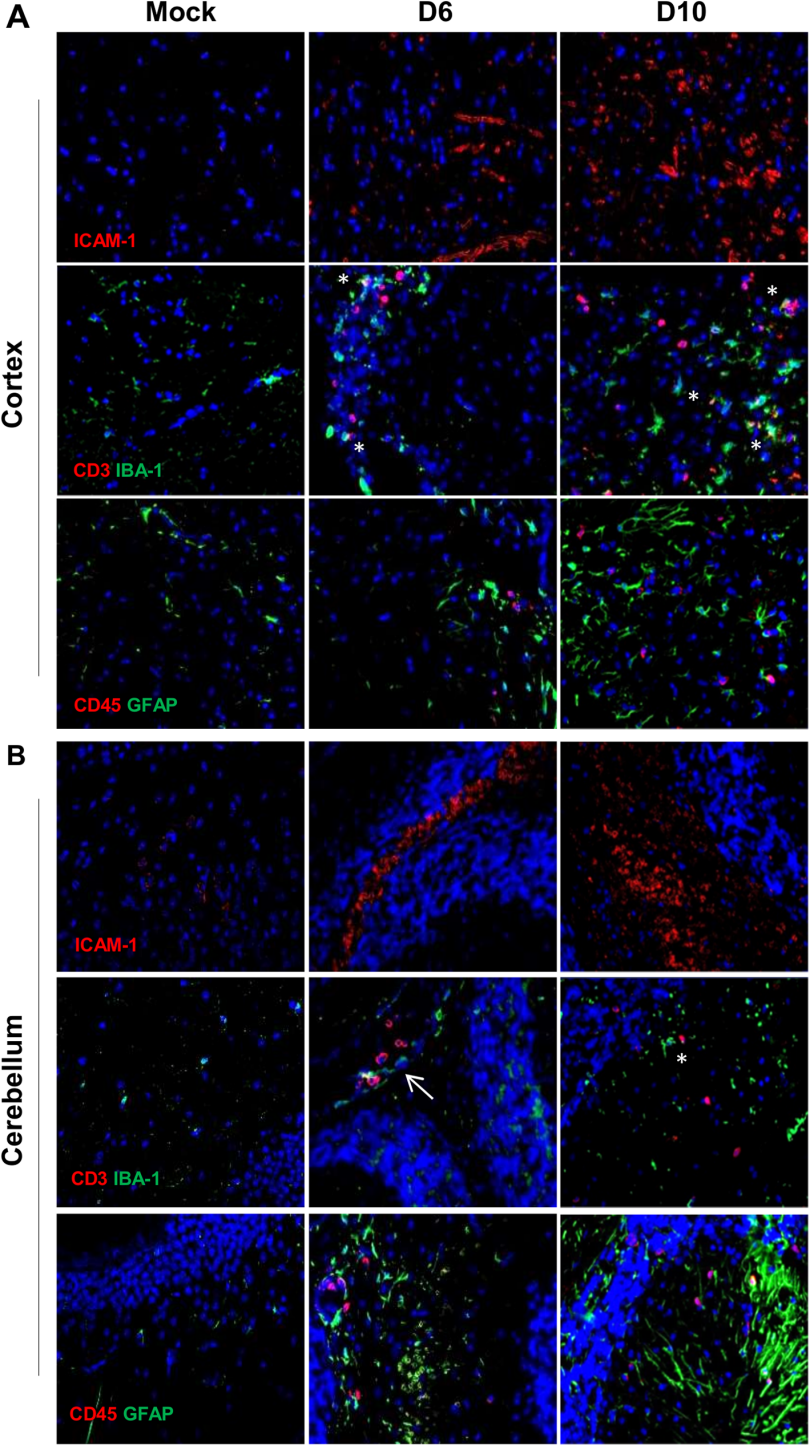
Vascular activation and cellular recruitment in *Orientia*-infected brains. Mice were inoculated with a lethal dose of *O*. *tsutsugamushi* or PBS, as described in *Fig 1*. Frozen cortex tissues **(A)** and cerebellum tissues **(B)** were fixed and stained with antibodies specific to ICAM-1, CD3, CD45 (red), IBA-1, and GFAP (green), respectively, as well as DAPI (blue, for nuclei). Representative images (photographed at 40X) are shown. Foci of T cell-phagocyte interactions are marked with asterisks. An arrow marks the blood vessel.

**Fig 5:**
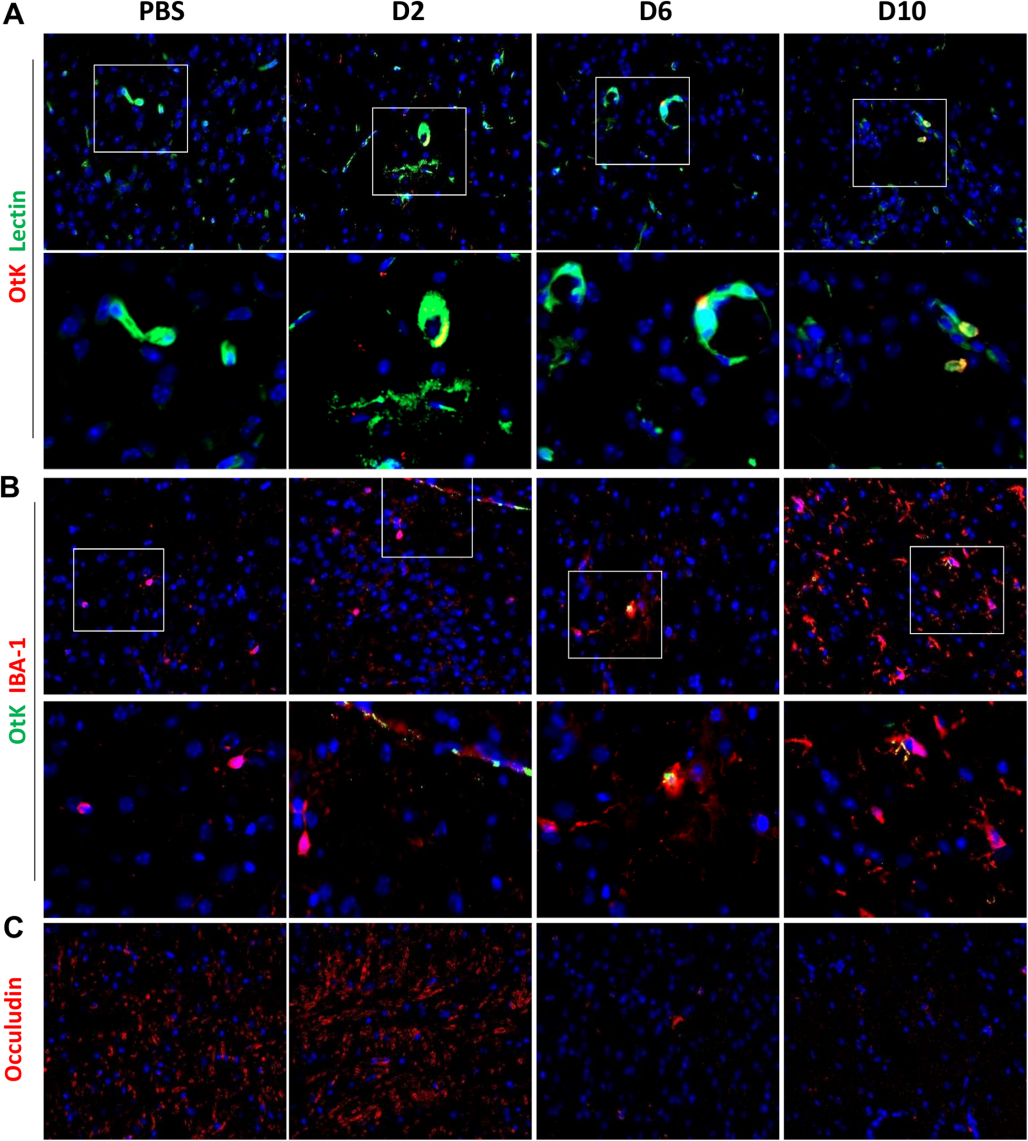
*Orientia* co*-*localization with brain endothelial cells and phagocytes during infection. Mice were inoculated with a lethal dose of *O*. *tsutsugamushi* Karp strain (OtK) or PBS, as in *Fig 1*. Frozen brain tissues were fixed and stained respectively with **A)** FITC-conjugated lectin (green) and rabbit anti-OtK (red), **B)** rat anti-IBA-1 (red) and rabbit anti-OtK (green), **C)** rat anti-occludin (red, for tight junction), as well as DAPI (blue). Shown are representative images (photographed at 40X), as well as their corresponding close-up views from the boxed areas.

Given the marked elevation of transcripts for proinflammatory cytokines (IFN-γ and TNF-α) and T-cell recruiting signals (CXCL9 and CXCR3) in infected brains (Figs 1 and 2), we decided to focus on lethal infection, especially vascular/cellular activation and leukocyte recruitment. PBS controls exhibited baseline staining for ICAM-1, as well as classical staining patterns for IBA1^+^ professional phagocytes and GFAP, a marker expressed exclusively in astrocytes in the CNS (**S4 Fig**); mice at day 2 of infection had no overt pathological changes. The ICAM-1-positive staining was strong in the cortex at day 6 and more intensified at day 10 (**Fig 4**), implying the activation of EC (and possibly other immune cells) during disease progression. The increased staining for CD3^+^ T cells and CD45^+^ leukocytes, especially at day 10, suggested to us an increased cellular recruitment and/or replication in the inflamed brains. Of note, there was an increased number, size, and foot-extrusion for IBA1^+^ phagocytes and GFAP^+^ astrocytes in the cortex and cerebellum, especially at day 10. The unique foot processes of IBA1^+^ and GFAP^+^ cells, as well as their close interactions with CD3^+^ T cells and/or CD45^+^ leukocyte (**asterisks**, Fig 4), were indicative of cellular activation and interactions in the CNS. To reveal bacterial distribution and vascular damage, we co-stained OtK with host cells. The co-localization of OtK in lectin^+^ EC (**Fig 5A**) and in IBA-1^+^ phagocytes (**Fig 5B**) was sparse but evident at days 2, 6, and 10, respectively. The loss of occludin staining at days 6–10 implied severe damage in the inter-endothelial junctions, as described in other infection models [23]. These immunostaining data were consistent with pathological evaluations (Fig 3) and qRT-PCR data (Fig 1), implying progressive and prominent vascular damage, cellular infiltration, and activation in focal lesions of mouse brains during lethal infection with OtK bacteria.

### Type 1-skewed, but type 2-imparied, immune responses in the lungs of *O*. *tsutsugamushi*-infected mice

**Fig 6:**
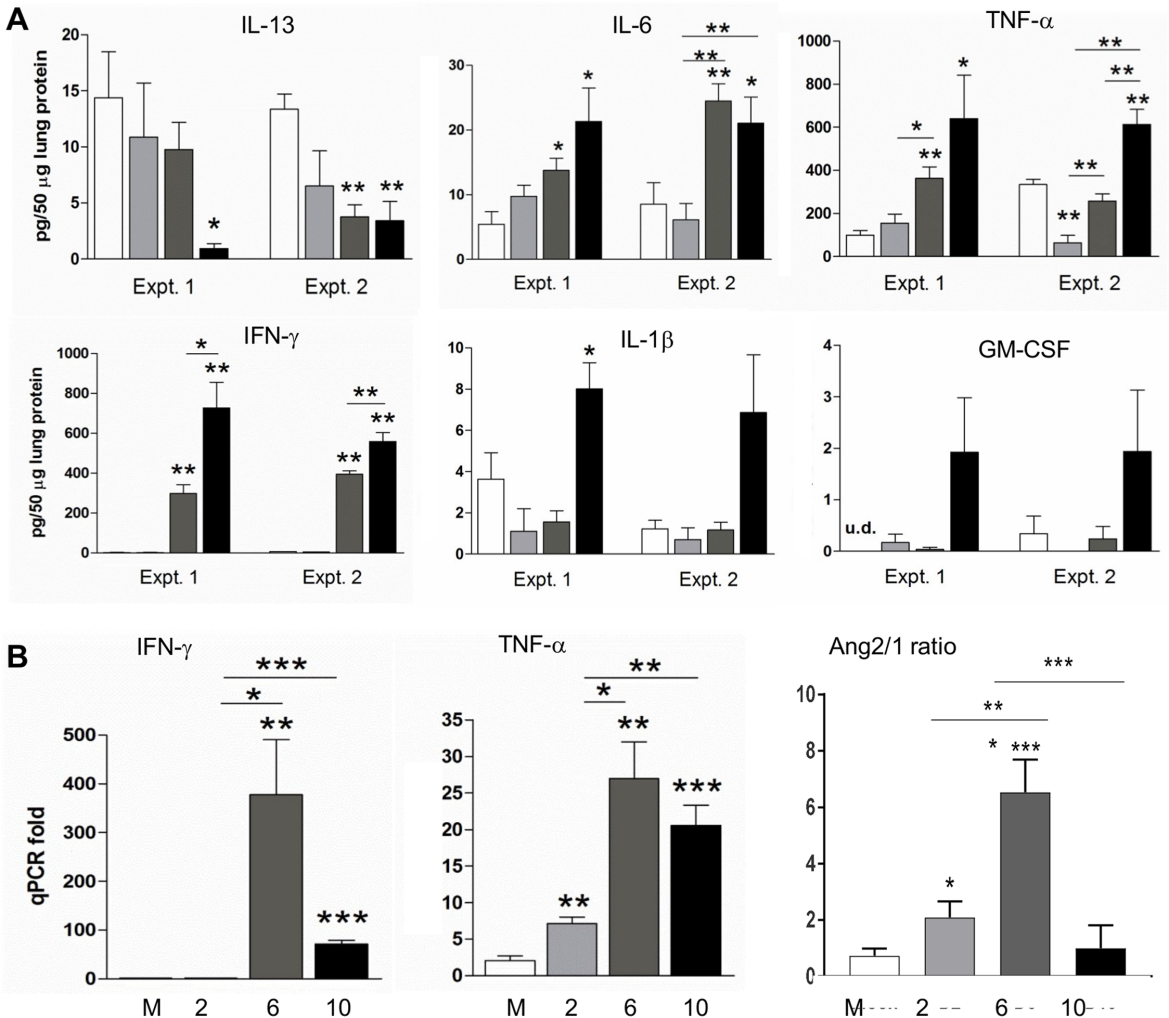
Type 1-skewed immune responses and vascular damage in *O*. *tsutsugamushi*-infected lung tissues. Mice (5/group) were inoculated i.v. with a lethal dose of bacteria or PBS (open bars), as in *Fig 1*. **A)** At 2, 6, and 10 days of infection (grey to black bars), lung tissue homogenates were measured for cytokine protein levels via Bioplex. Shown are data from two independent experiments. **B)** Total RNAs were extracted from lung tissues for qRT-PCR analyses. Data are presented as mean in each group and are presented as “qPCR fold” after normalization to housekeeping gene (GAPDH). Representative data from three independent studies with similar trends are shown. *, *p* < 0.05; **, *p* < 0.01, ***, *p* < 0.001 (compared with mock groups, or the marked groups).

CNS alterations are evident in scrub typhus patients [7,8,9], in a mouse self-limiting model [15], and in our studies herein. However, *Orientia* antigens in mouse brains seems sparse (Fig 5)[17], often 50-200-fold less in bacterial gene copies than in the lungs [15,17]. Given that lungs are the primary target for OtK infection and immune alterations [17,18], we speculated that systematic, proinflammatory responses that damage the brain blood barrier can markedly exacerbate pathological changes in the CNS. To support this notion, we focused on lethal infection and examined protein levels of key cytokines in lung tissue homogenates by using a Bioplex assay. Samples from two independent experiments revealed a significant, infection time-dependent suppression of IL-13, a classical type 2 cytokine, especially at the late stages (day 10) of infection (**Fig 6A**). Meanwhile, there was a time-dependent production of proinflammatory cytokines in infected lung tissues. Compared with the mock controls, day 10 samples showed approximately 2–3 fold increase in IL-6, 2–6 fold increase in TNF-α, but 500–700 fold increase in IFN-γ, respectively. The trends of increase in IL-1β and GM-CSF, as well as IL-18, IL-27, and IL-10 (**S5 Fig**), were observed, but their statistical significances were marginal, either due to their relatively low protein abundance and/or to the sample-to-sample variations. There were no major changes in the levels of IL-2, IL-5, IL-12p70, and IL-23; several other markers in the Bioplex kit (IL-4, IL-9, IL-17, and IL-22) were not detectable in lung tissue homogenates. To validate these results and expand the findings, we examined mRNA levels of several hallmark genes in the same lung tissues. We found a relatively low, but statistically significant, elevation of TNF-α and Ang2/Ang1 ratio at day 2 (**Fig 6B**). The mRNA levels of IFN-γ, TNF-α, and Ang2/Ang1 ratio reached their peak at day 6, with an approximately 360-fold, 10-fold, and 5-fold increase, respectively, some of which were sustained to day 10. Collectively, these data from infected lungs were consistent with previous reports from our and other groups [18,24,25], implying an early and profound type 1-skewed inflammatory responses in the lungs and in macrophages that may influence the outcome of infection in other organs.

## Discussion

In this study, we presented new evidence for type 1-skewed neuroinflammation, foci of vascular leakage, and activation of macrophages/astrocytes during *O*. *tsutsugamushi* infection and suggested their association with pathogenesis and lethality. Specifically, our data revealed that i.v. inoculation with lethal and sublethal doses of OtK resulted in significant expression of TLR2/TLR4/TLR9 genes and triggered potent type 1-skewed immune responses in inflamed brain. These gene expression levels correlated with leukocyte influx and macrophage/astrocyte activation in the cortex or cerebellum. Our data support and extend previous reports in mouse models [15,17], providing additional lines of evidence for cellular responses in the CNS, as well as new clues for further investigation of tissue-specific immune mechanisms during *O*. *tsutsugamushi* infection.

The roles of specialized pattern recognition receptors (PRRs) during *O*. *tsutsugamushi* infection remain elusive. Intracellular PRRs, including nucleotide-binding oligomerization domain-containing protein 1 (NOD1)-related pathway, are known to contribute to inflammasome activation in *O*. *tsutsugamushi*-infected EC and IL-1β production from infected macrophages [26,27]. However, unlike other Gram-negative bacteria or closely related bacteria from the genus *Rickettsia*, TLR4 and TLR2 seem to play no or limited roles in the uptake of or defense against *Orientia*, due to the lack of lipopolysaccharide and peptidoglycan in its cell wall [28]. A recent report has shown that TLR2 deficiency had no impact on the i.d. route of OtK infection in C57BL/6 mice; however, i.p.-inoculated TLR2^-/-^ mice were more resistant to lethal infection than wild-type controls, with major differences only at late/convalescence phases [29]. In this study, we found a significant and consistent increase in TLR2, TLR4, and TLR9 transcripts in the brains of lethally infected mice at days 6 and 10 (Fig 1), as well as in brains of sublethal infection (Fig 2). These TLR expression trends were correlated with the up-regulation of proinflammatory molecules (IFN-γ, TNF-α, CXCL9, and CXCR3) and vascular stress markers (Ang2, Ang2/1 ratio). Although this study did not examine the ligands for TLR upregulation, it is known that *O*. *tsutsugamushi* contains heat-stable components as a TLR agonist [29]. Also, infection-triggered release and process of host damage-associated molecular pattern (DAMP) molecules can induce TLR upregulation and exacerbate cytokine/chemokine production. Along this line, we have reported that IL-33, a nuclear protein released from OtK-infected mouse tissues or human ECs, can serve as alarmin-like DAMPs and promote inflammation and tissue damage [19]. Among other possible DAMPs, high-mobility group box protein 1 (HMGB1, a non-histone DNA-binding protein and a known TLR2/TLR4/TLR9 ligand [30,31,32,33]), as well as histone proteins (known TLR9 ligands), warrants further investigation. Recent reports have revealed the roles of IL-33 in CNS injury and post-injury recovery, as well as a role for HMGB1 in CNS injury, in humans and mouse models [34,35,36,37,38]. It will be interesting to examine whether these DAMPs contribute to TLR2/TLR4/TLR9 up-regulation in OtK-infected brains.

Regardless of innate immune recognition mechanisms, CNS injury and cellular activation were evident and further defined in our infection models. To date, there have been no detailed immunological studies that focused on the brain during *Orientia*-infection in an animal model. Our findings of glial nodules consisting of perivascular infiltration by lymphocytes and macrophages in the neuropil, as well as perivascular hemorrhage (Figs 3 and 4), resemble the lesions and cell tropism described in humans [39,40]. Furthermore, we provided new evidence for increased detection of ICAM-1^+^ vascular cells, CD3^+^ T cells, CD45^+^ leukocytes, IBA1^+^ phagocytic cells, and GFAP^+^ astrocytes in the cortex and cerebellum of OtK-infected mice, especially at day 10 (Fig 5). These pathological changes correlated with up-regulated proinflammatory markers (IFN-γ, TNF-α, CXCL9, and CXCR3), implying an increased leukocyte recruitment, macrophage/astrocyte activation, and cell-cell interactions in inflamed brains. Our finding of a gradual increase of CXCR3 transcripts in the brains of lethal infection (Fig 1), but significantly higher expression levels in the brains of sublethal infection (Fig 2), is intriguing and warrants further investigation, as persistent infection can occur in human patients [2,41] and mouse models [16,17,19]. Similar to mouse models of rickettsial and viral infections, in which CXCR3^+^CD8^+^ T cells and other immune cells contribute to pathogen clearance and pathogenesis [42,43,44,45], CXCR3 and its ligands may play complex roles in OtK-induced neuroinflammation, depending on the infection doses and disease stages. While tightly regulated cellular activation and NO production in the brain and other organs promote self-healing following sublethal infection with OtK [15,16], we believed that OtK-mediated cell death and uncontrolled type-1 inflammation collectively lead to rapid and progressive loss of Tie2 and tight junctions and endothelial barrier function, leading to host death (Figs 1 and 5). Our findings are in agreement with other studies for CNS involvement in viral infections [46]. Together, our data of OtK localization in EC and macrophages supported and extended observations made from patients [14], as well as conclusions made from previous animal studies from us and other groups [15,17]. Our detection of sparse OtK bacteria in the brains suggests an important role for immune-mediated vascular activation and damage during disease progression.

This hypothesis was supported by our analyses of cytokine protein levels in lung tissues. Given that pulmonary involvement is a well-documented complication of human scrub typhus [39], and that lungs are the major target of OtK replication regardless of infection routes [15,16,17], it was logical to identify cytokine protein profiles via Bioplex. We consistently detected a marked reduction in IL-13, but significant elevation of IL-6, TNF-α, and IFN-γ at days 6 and 10 (Fig 6). IL-1β and GM-CSF proteins were less or not significant at day 10, but their trends correlated with disease progression and mortality, as in our previous studies [16]. Of note, lung up-regulation of TNF-α (Fig 6), IL-1β, IL-6, and IFN-γ [18] were significant at day 2; all of these markers reached their expression peak at day 6. This early and strong induction of type 1-skewed inflammatory responses was likely triggered by bacterial replication in the lungs [18], leading to early and severe EC damage in the lungs (Fig 6B). It is worth mentioning that OtK-infected lungs can share common immunological features with other organs (liver, spleen, kidneys, etc.) [18], but also can display tissue-specific hallmarks [19]. Multivariate analyses from human patients also reveal a positive association between the presence of pneumonitis and the occurrence of scrub typhus meningitis and meningoencephalitis [47]. At present, it remains unclear how these lung responses influence neuroinflammation, but it has been shown that cytokine-mediated STAT3 activation can down-regulate occludin levels and increase endothelial permeability through the induction of VEGF production in EC [48]. Therefore, it will be important to further examine the molecular mechanisms underlying lung-brain infection and tissue damage and how such changes impact the release of proinflammatory cytokines and endogenous danger signals. Identification of tissue-released pathogenic biomarkers and validation of their association with scrub typhus severity are of value for the management of this neglected infectious disease.

In sum, this study has provided the first critical insight into brain gene expression profiles during lethal and sublethal OtK infections in C57BL/6 mice. The most important findings in this study are the progressive up-regulation of certain TLR molecules and type-1 cytokines, as well as marked alterations in the Ang/Tie2 axis and loss of tight junctions, in infected brain tissues. The alterations in the Ang/Tie2 axis are likely to be pathogenic hallmarks in OtK-infected brains and lungs, and possibly in other major organs of OtK infection. The immunofluorescent staining data have also validated and expanded our understanding on *Orientia* bacterial localization in severe scrub typhus cases [40,49]. Given the relatively low bacterial loads in the brains and the complex contribution of cytokines/chemokines to neuroinflammation [50], it will be important to further examine the pathophysiological mechanisms underlying CNS involvement and immune-mediated vascular activation in severe scrub typhus.

## Supporting information

S1 Table Real-time PCR primers for mouse genes studied herein. (DOCX)

S1 Fig Progression of disease following infections with a lethal or sub-lethal dose of *O*. *tsutsugamushi* Karp strains in C57BL/6 mice. A) Body weight change for two separate experiments (5/group) following lethal infection. B) Body weight change (%) and survival curves for mice with sublethal versus lethal infections.
(TIF)

S2 Fig Quantification of lesion foci in brains of sub-lethal versus lethal infection. Mice were inoculated with *O*. *tsutsugamushi* Karp strain or PBS, as described in *Fig 2*. Brain tissues were collected at day 10 for lethal infection and day 12 for sublethal infection, fixed, and stained, as in *Fig 3*. For each brain sample, 10 microscopic images were numerically assessed for total number of lesions per 10X field-of-view. The averaged data are presented as “number of lesions per animal.” Groups were statistically compared using the Student’s t-test. ##, *p* < 0.01, ####, *p* < 0.0001 (compared with the mock groups). ****, *p* < 0.0001 (compared with marked groups).
(TIF)

S3 Fig Pulmonary congestion and edema following sublethal and lethal infections. Mice were inoculated i.v. with *O*. *tsutsugamushi* Karp strain or PBS, as described in *Fig 2*. Lung samples were collected from the mock, sublethal groups (day 12), and lethal groups (day 10), fixed in 10% formalin, and embedded in paraffin. Sections were stained with H&E. Images were photographed at 10X (A, C, E) or at 40X (B, D, F), respectively. The boxed areas represent the close-up view at 40X, and the circle highlights cellular infiltration and tissue damage.
(TIF)

S4 Fig *Orientia*-infected, but not control, brains with vascular activation and cellular recruitment. Mice were inoculated with a lethal dose of *O*. *tsutsugamushi* or PBS, as described in *Fig 1*. Frozen brain tissues of mock and day 10 groups were stained with antibodies specific to ICAM-1, CD3, CD45 (red), IBA-1 and GFAP (green), as well as with DAPI (blue, for staining nuclei), respectively. Shown are single-staining versus merged representative images (photographed at 40X).
(TIF)

S5 Fig Mice were inoculated i.v. with a lethal dose of bacteria or PBS (open bars), as in Fig 1. At 2, 6, and 10 days of infection (grey to black bars), lung tissue homogenates were measured for cytokine protein levels via Bioplex. Shown are data from two independent experiments.
(TIF)
