# Supplementary material for: Type 1-skewed neuroinflammation and vascular damage associated with Orientia tsutsugamushi infection in mice
Source: PLoS Negl Trop Dis. 2017 Jul 24;11(7):e0005765. doi: 10.1371/journal.pntd.0005765 (PMC5542690; doi:10.1371/journal.pntd.0005765)
Supplement: S1 Table — (DOCX) [file pntd.0005765.s001.docx]

**Supplement Table 1. Real-time PCR primers for mouse genes studied herein.**

| **Gene** | Forward (5’ to 3’) | Reverse (5’ to 3’) |
| --- | --- | --- |
| TLR2 | CACCACTGCCCGTAGATGAAG | AGGGTACAGTCGTCGAACTCT |
| TLR4 | AAATGCACTGAGCTTTAGTGGT | TGGCACTCATAATGATGGCAC |
| TLR9 | ACAACTCTGACTTCGTCCACC | TCTGGGCTCAATGGTCATGTG |
| CXCR3 | TACCTTGAGGTTAGTGAACGTCA | CGCTCTCGTTTTCCCCATAATC |
| Tie2 | CTGGAGGTTACTCAAGATGTGAC | TCCGTATCCTTATAGCCTGTCC |
