# Supplementary figures and images for: Type 1-skewed neuroinflammation and vascular damage associated with Orientia tsutsugamushi infection in mice
Source: PLoS Negl Trop Dis. 2017 Jul 24;11(7):e0005765. doi: 10.1371/journal.pntd.0005765 (PMC5542690; doi:10.1371/journal.pntd.0005765)

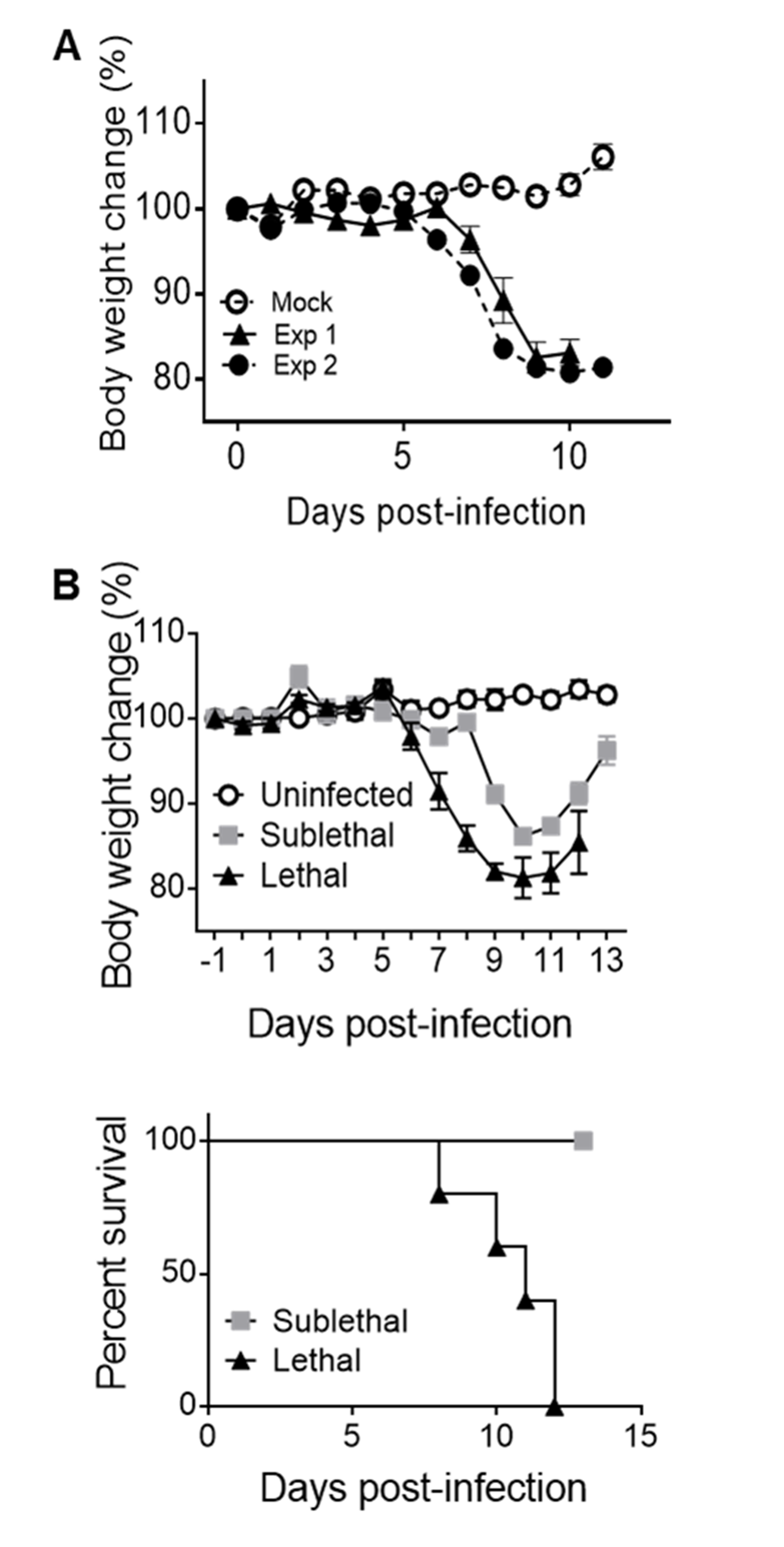

Supplement: S1 Fig — A) Body weight change for two separate experiments (5/group) following lethal infection. B) Body weight change (%) and survival curves for mice with sublethal versus lethal infections. (TIF) [file pntd.0005765.s002.tif]

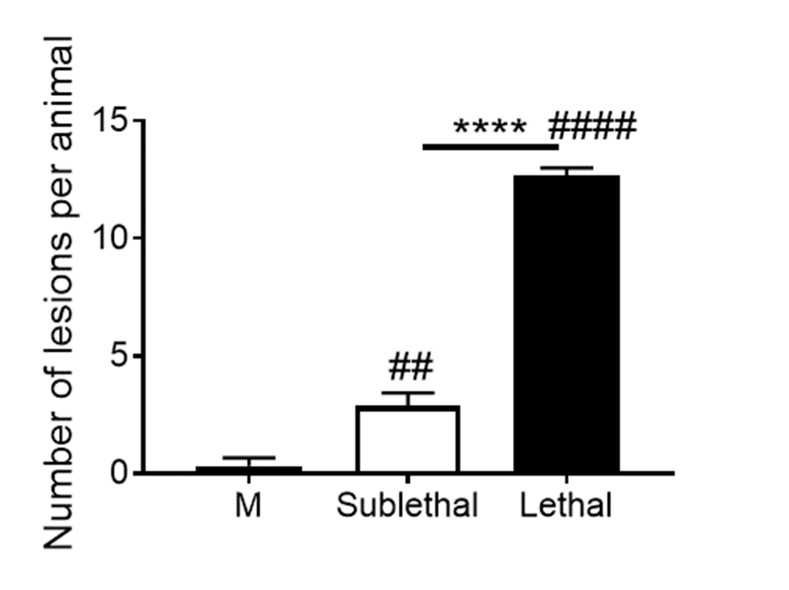

Supplement: S2 Fig — Mice were inoculated with O. tsutsugamushi Karp strain or PBS, as described in Fig 2. Brain tissues were collected at day 10 for lethal infection and day 12 for sublethal infection, fixed, and stained, as in Fig 3. For each brain sample, 10 microscopic images were numerically assessed for total number of lesions per 10X field-of-view. The averaged data are presented as “number of lesions per animal.” Groups were statistically compared using the Student’s t-test. ##, p < 0.01, ####, p < 0.0001 (compared with the mock groups). ****, p < 0.0001 (compared with marked groups). (TIF) [file pntd.0005765.s003.tif]

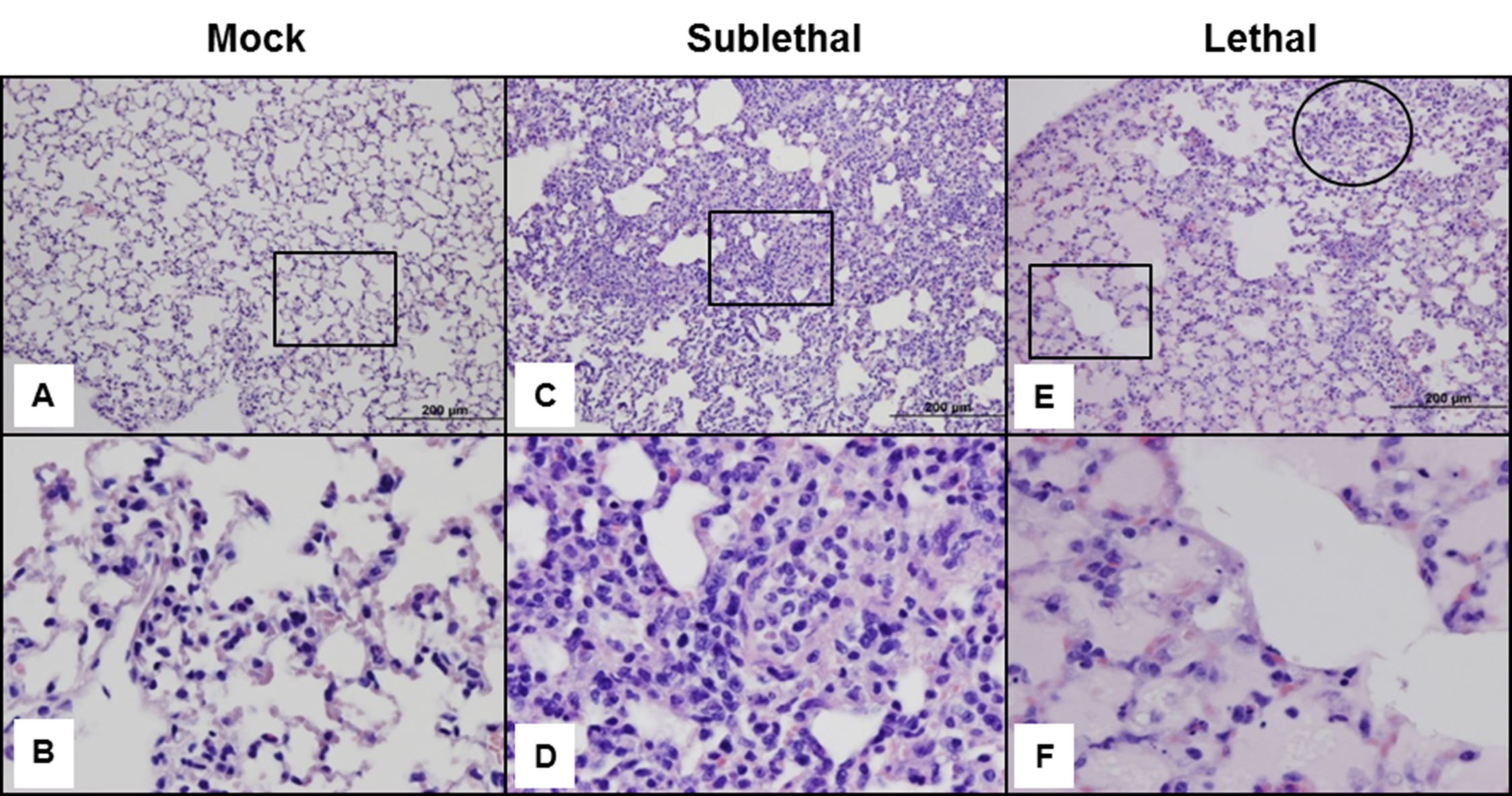

Supplement: S3 Fig — Mice were inoculated i.v. with O. tsutsugamushi Karp strain or PBS, as described in Fig 2. Lung samples were collected from the mock, sublethal groups (day 12), and lethal groups (day 10), fixed in 10% formalin, and embedded in paraffin. Sections were stained with H&E. Images were photographed at 10X (A, C, E) or at 40X (B, D, F), respectively. The boxed areas represent the close-up view at 40X, and the circle highlights cellular infiltration and tissue damage. (TIF) [file pntd.0005765.s004.tif]

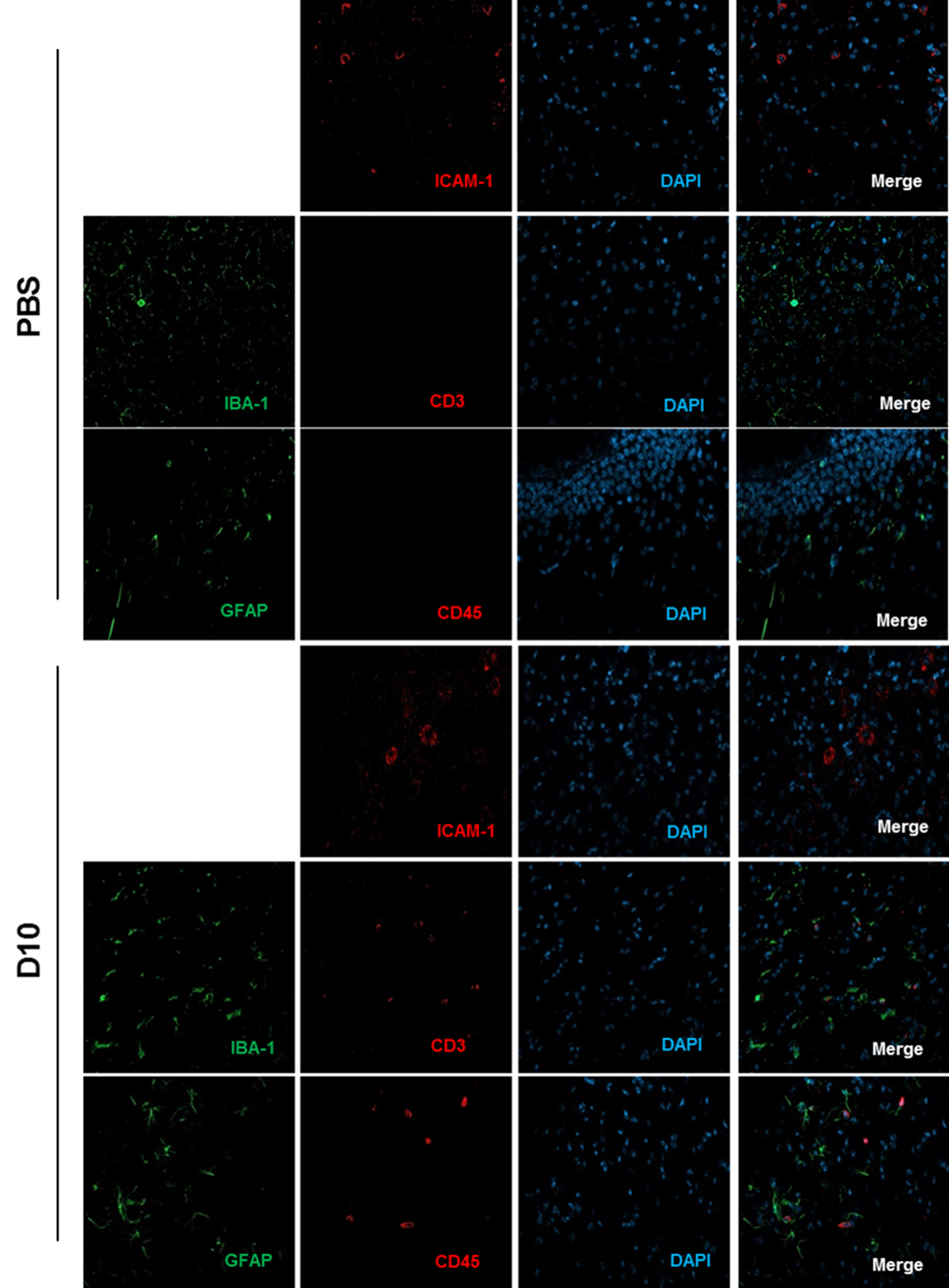

Supplement: S4 Fig — Mice were inoculated with a lethal dose of O. tsutsugamushi or PBS, as described in Fig 1. Frozen brain tissues of mock and day 10 groups were stained with antibodies specific to ICAM-1, CD3, CD45 (red), IBA-1 and GFAP (green), as well as with DAPI (blue, for staining nuclei), respectively. Shown are single-staining versus merged representative images (photographed at 40X). (TIF) [file pntd.0005765.s005.tif]

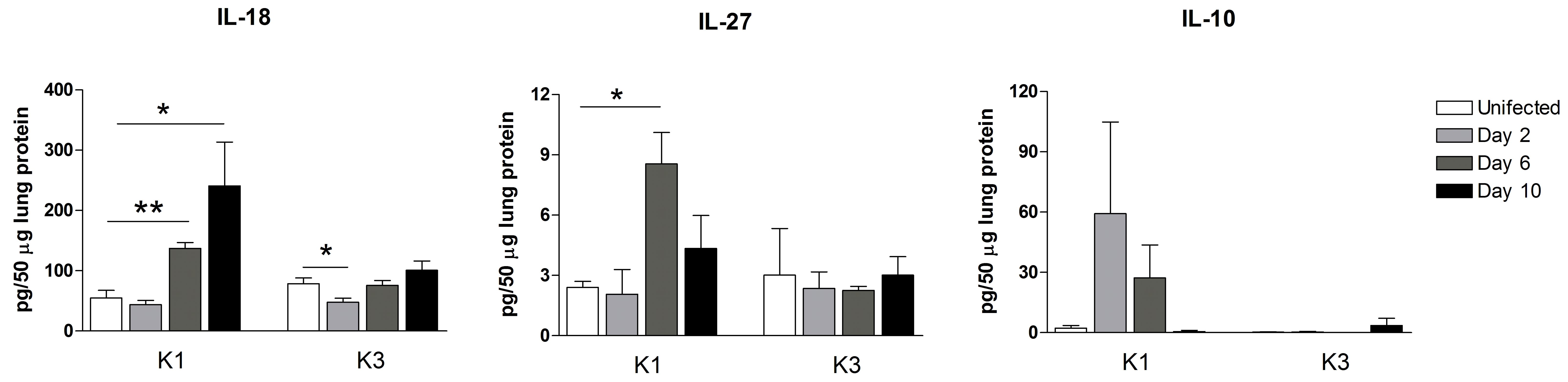

Supplement: S5 Fig — At 2, 6, and 10 days of infection (grey to black bars), lung tissue homogenates were measured for cytokine protein levels via Bioplex. Shown are data from two independent experiments. (TIF) [file pntd.0005765.s006.tif]
